# Elevated Expression of the Serine-Arginine Protein Kinase 1 Gene in Ovarian Cancer and Its Role in Cisplatin Cytotoxicity *In Vitro*

**DOI:** 10.1371/journal.pone.0051030

**Published:** 2012-12-07

**Authors:** Kunle Odunsi, Paulette Mhawech-Fauceglia, Christopher Andrews, Amy Beck, Olajumoke Amuwo, Shashikant Lele, Jennifer D. Black, Ruea-Yea Huang

**Affiliations:** 1 Department of Gynecologic Oncology, Roswell Park Cancer Institute, Buffalo, New York, United States of America; 2 Department of Immunology, Roswell Park Cancer Institute, Buffalo, New York, United States of America; 3 Department of Pathology and Laboratory Medicine, Roswell Park Cancer Institute, Buffalo, New York, United States of America; 4 Department of Biostatistics, State University of New York at Buffalo, Buffalo, New York, United States of America; 5 Department of Pharmacology and Therapeutics, Roswell Park Cancer Institute, Buffalo, New York, United States of America; 6 Center for Immunotherapy, Roswell Park Cancer Institute, Buffalo, New York, United States of America; Cedars-Sinai Medical Center, United States of America

## Abstract

Alternatively spliced variants of several oncogenes and tumor suppressors have been shown to be important for their tumorigenicity. In the present study we have tested whether serine-arginine protein kinase 1 (SRPK1), a major regulator of splicing factors, is involved in ovarian cancer progression and plays a role in chemo-sensitivity. By Western blot analyses, SRPK1 protein was found to be overexpressed in 4 out of 6 ovarian cancer cell lines as compared with an immortalized ovarian surface epithelial cell line; and in 55% of ovarian tumor samples as compared with non-neoplastic ovarian tissue samples. Reduction of SRPK1 expression using small interfering RNA (siRNA) encoding small hairpin RNA in ovarian cancer cells led to (i) reduced cell proliferation rate, slower cell cycle progression and compromised anchorage-independent growth and migration ability *in vitro*, (ii) decreased level of phosphorylation of multiple serine-arginine proteins, and P44/42MAPK and AKT proteins, and (iii) enhanced sensitivity to cisplatin. Together, these results suggest that elevated SRPK1 expression may play a role in ovarian tumorigenesis and SRPK1 may be a potential target for ovarian cancer therapy.

## Introduction

It has been estimated that 35–59% of human genes are alternatively spliced, which contributes greatly to the complexity of human cellular functions [1], [2]. It is therefore not surprising that the activities of some oncogenes and tumor suppressor genes are modulated by alternative splicing [3], [4]. For example, aberrant alternative splicing of certain tumor suppressors, such as breast cancer 1 and 2 (BRCA1/2) [5], Wilms tumor 1 (WT1) [6], and adenomatous polyposis coli (APC) [3], results in mutations that account for inherited and sporadic susceptibility to cancer. In addition, certain splicing factors have been found to be overexpressed in tumors and have been implicated as onco-proteins [7], [8].

SRPK1 (serine-arginine protein kinase 1) belongs to the SR kinase family of proteins and regulates the SR family of splicing factors. The SR splicing factors are some of the auxiliary proteins that are required for pre-mRNA splicing in mammalian cells. SR proteins are characterized by one or two RNA recognition motifs at the N terminus and a SR-rich domain at the C terminus [9], [10], [11]. The SR domain is thought to promote protein-protein interactions during assembly [12]. SR proteins are regulated by reversible phosphorylation mediated by SRPK1. While phosphorylated SR proteins are required for initiating spliceosome assembly at the earliest stage, dephosphorylation is essential for splicing to take place in the spliceosome [13], [14]. Evidence has shown that both hypo- and hyper-phosphorylation of SR proteins are detrimental to splicing [14], [15]. Thus, the level of SRPK1 is crucial for maintaining the proper balance between phosphorylated and dephosphorylated SR proteins. Overexpression of SRPK1 protein has been documented in acute type Adult T-cell leukemia [16], chronic myelogenous leukemia [17] pancreatic, breast, and colon cancers [18], [19], [20]. Interestingly, SRPK1 is expressed in adult male germ cells, but generally not in most other normal adult tissues, suggesting a cancer/testis-like distribution [16], [21]. Together, these studies suggest that SRPK1 is likely to play an important role in cancer development.

We [22] and others [23] have previously found that inactivation of *SKY1*, the yeast homologue of the splicing factor SRPK1, enhances resistance of yeast cells to cisplatin (cDDP). However, whether SRPK1 expression plays a role in determining sensitivity or resistance of human tumors to chemotherapy remains unclear for the following reasons. First, lower level of SRPK1 expression has been shown to correlate with resistance of ovarian [23], testicular [20] and HT29 [24] tumor cell lines to platinum-containing treatment regimens. Second, it has been recently shown that disruption of SRPK1 expression by siRNA increases apoptosis caused by cDDP in pancreatic, colon and breast cancer cell lines [18], [19].

In the present study we sought to examine whether SRPK1 expression is associated with ovarian cancer progression, whether the expression pattern of SRPK1 correlates with clinical responses to treatment involving cDDP and whether inhibition of SRPK1 alters the sensitivity ovarian cancer cells to cDDP. First, we found that elevated SRPK1 protein level was present in approximately 55% of ovarian tumor samples as compared with non-neoplastic ovarian tissue samples. Second, siRNA-mediated inhibition of SRPK1 led to reduced OVCa cell proliferation rate, *in vitro* cell migration, tumorigenic potential and slower cell cycle progression. These phenotypes were associated with SRPK1-mediated alterations of MAPK/AKT signaling pathways, since the levels of phosphorylated (activated) MAPK/AKT protein were reduced in the SRPK1 knockdown cells. Finally, in contrast to the yeast system, we made the surprising observation that inhibition of SRPK1 enhanced sensitivity to cDDP treatment, suggesting that SRPK1 may be a target for therapy of ovarian cancer.

## Materials and Methods

### Ethics Statement

The study involving human subjects was conducted under a protocol approved by the RPCI Institutional Review Board (CIC0215). All tissue specimens were collected from patients who provided written informed consent.

### Patients and Ovarian Tumor Specimens

Flash frozen tissue specimens (n = 47) were obtained from patients undergoing debulking surgery for epithelial ovarian cancer at the Roswell Park Cancer Institute (RPCI), Buffalo, NY between 1995 and 2006. Normal ovarian samples (n = 9) were obtained from patients undergoing hysterectomies for benign conditions such as leiomyoma. Clinicopathologic information for the entire cohort, including response to chemotherapy, is maintained in a database in the Department of Gynecologic Oncology.

### Cell Culture

Ovarian cancer cell lines SKOV3 and OVCAR3 were obtained from the American Type Culture Collection (ATCC; Manassas, VA). A2780 and A2008 cells and their cDDP-resistant counterparts A2780/CP [25] and A2008/C13 [26] cells were obtained from Dr. Steven Howell (University of California, San Diego). These cells were maintained in RPMI1640 medium supplemented with 10% fetal bovine serum (FBS). A non-transformed ovarian surface epithelial cell line (IOSE-385, hereafter designated as IOSE) was immortalized with the SV40 early genes [27] and obtained from Dr. Nelly Auersperg (University of British Columbia, Canada). IOSE cells were maintained in M199/MCDB 105 medium (Sigma-Aldrich, St. Louis, MO) supplemented with 5% FBS and gentamycin (Invitrogen, Carlsbad, CA).

### shRNA and rescue-SRPK1 Constructs

shSRPK1-1 and shSRPK1-2, encoding shRNA targeting nucleotides 288 to 308 (CAAGAAGATCCTAATGATTA) and 1423 to 1443 (GGTCAGTCATTCAGTGAACAA), respectively, of the SRPK1 mRNA, were purchased from Open Biosystems (Huntsville, AL). For transfection, SKOV3 and A2780/CP cells were seeded in 6-well plates to 80% confluency and transfected with 3–4 µg of plasmid DNA using Lipofectamine 2000 (Invitrogen, Carlsbad, CA). RNA and proteins from control or shSRPK1-transfected cells were analyzed 3 days after transfection. Stable shSRPK1 clones were selected using 2 µg/ml of puromycine for 3 weeks. For SRPK1-rescue plasmid, a primer containing 3 silenct mutations (GCAAGAAGATCCTAATGATTA, underline nucleotides were changed to G, G, C, respectively) within the shSRPK1-1 target sequences was introduced into p-CMV-flag-SRPK1 plasmid (a gift from Dr. Xiang-Dong Fu, University of California, San Diego) using asymmetric PCR [28]. Transfection was performed as described above and stable cones were selected using G418 (Invitrogen, Carlsbad, CA).

### Chemicals

Cisplatin (cDDP, *cis*-diammine-dichloro-platinum II) and MTT {3-(4,5-dimethylthiazol-2-yl)-2,5-diphenyltetrazolium bromide} were purchased from Sigma-Aldrich (St. Louis, MO). Stock cDDP solution was prepared in DMSO (330 mM), stored as aliquots at −20°C, and used within 2 weeks. cDDP was further diluted in medium before adding to the cells.

### Western Blot Analysis

Twenty to thirty milligrams of frozen tumor tissue was homogenized with a mortar and pestle in tissue sample buffer (10% SDS, 5% β-mercaptoethanol, 50 mM Tris-HCl, pH 8.0, 10% glycerol) for protein extraction. Cell lysates were extracted using RIPA buffer (150 mM NaCl, 1% NP-40, 0.5% deoxycholic acid, 0.1% SDS, 50 mM Tris-HCl, pH 8.0). Proteins were resolved by 10% SDS-PAGE, transferred to PVDF membrane (Milipore, Billerica, MA) and blocked in TBS containing 0.05% Tween-20 and 5% dried milk overnight at 4°C. Blots were incubated with primary antibody for 1–2 hr, and then with peroxidase-conjugated secondary antibody for 1 hr at room temperature. Following incubation, membranes were washed repeatedly and proteins were visualized using the ECL (enhanced chemiluminescense) kit (Pierce, Rockford, IL). Signal was digital photographed and quantified using densitometry with an Alpha imager (Alpha Innotech, San Leandro, CA). Antibodies used to detect the SRPK1 was from BD Biosciences (San Jose, CA), phosphorylated SR proteins were mAB104 [29] isolated from hybridoma cells (ATCC, Manassas, Virginia), total p44/42 MAPK and AKT (AKT1/2/3, sc-8312) were from Cell Signaling (Danvers, MA) and Santa Cruz, respectively, phosphorylated MAPKp42/44 (Thr^202^/Tyr^204^) and AKT (Ser^473/^Thr^308^) were from Cell Signaling (Danvers, MA), Antibody against Upf1 was a gift from Dr. Harry Dietz (Johns Hopkins University). Anti-actin was from Sigma-Aldrich (St. Louis, MO). Data were expressed as relative fold expression over actin.

### Immunohistochemical Staining

Sections (4 µm thick) from formalin-fixed, paraffin-embedded normal ovary and tumor tissue were processed for IHC as described previously [30]. Endogenous peroxidase was blocked with 0.3% hydrogen peroxidase for 30 min. Antigen retrieval was carried out in high pH buffer in a steamer for SRPK1 antibody (BD Biosciences). For negative control, the mouse-IgG was used.

### Reverse-transcription Polymerase Chain Reaction (RT-PCR) Analysis

Total RNA was extracted from ten million cells using Trizol reagent (Invitrogen) according to the manufacturer's instructions. cDNA was synthesized from 2 µg total RNA in 20 µl reaction buffer using M-MuLV reverse transcriptase, Ribolock ribonuclease Inhibitor (Fermentas Life Science, Glen Burnie, MD) and random hexamer primers after DNaseI digestion. The cDNA products were used for semi-quantitative PCR using Taq DNA polymerase (Fermentas Life Science, Glen Burnie, MD) The primers for SRPK1 (SRPK1-R, 5'-TGTTGTCCAGTGGTCCGTTA, and SRPK1-exon10, 5'- CAAGAAAAACTTGAAGAGTC) were designed according to the NCBI reference sequences. GAPDH (glyceraldehyde 3-phosphate dehydrogenase) was used as reference target sequence (primers: 5'-GAAGGTGAAGGTCGGAGTC and 5'- GAAGATGGTGATGGGATTTC).

### Clonogenic Survival Assay

Cells (3×10^2^) were seeded in 6-well plates overnight and treated with cDDP for 24 hr. After removing the drug, cells were washed with PBS and re-fed with drug-free medium and incubated for 10–14 days. Colonies were stained with 0.1% crystal violet and counted. Percentage cell survival is expressed relative to untreated control.

### MTT Assay

Cells (5×10^3^) were seeded in 96-well plates in triplicate overnight and treated with cDDP for 72 hr, MTT was added for 4 hr, and the formazan dye was dissolved with DMSO and read at 540 nm in a FL6000 microplate reader (BIO-TEK Instruments, Winooski, VT). Percentage cell survival is expressed relative to untreated control.

### Anchorage-independent Growth Assay

Cells (5×10^3^) were seeded in triplicate in 6-well plates containing a top layer of 0.3% soft agar and a 0.5% agar base. Twenty-four hour later, the average number of cells seeded per field was determined by counting cells in 5 different fields under the light microscope. Colonies formed (>0.1 mm in diameter) after 3 weeks of growth in soft agar were counted; 10 different fields were quantified per well and the average number of colonies per field was calculated. The AIG (anchorage-independent growth) index was expressed relative to the number of cells seeded.

### Wound Healing (Scratch) Assay

Cells were seeded in 60-mm plates to confluence and the cell monolayer was scraped in three straight lines with a 200-µl pipette tip to create “scratches”. Debris was removed with PBS and then the culture was re-fed with fresh medium. Photohgraphs of the wound area were taken at 0 and 28 hr after the scratches to calculate the cell migration rate. Twenty random measurements were taken for each time point. The relative migrating distances of the wound areas were measured on the images.

### Cell Cycle Analysis

Cells were synchronized at G2/M phase by treatment with nocodazole (600 ng/ml) for 12 hr, washed two times with PBS and then released into medium without drug. Cells were also synchronized at G0/G1 by placing in serum-free medium for 48 hr. The quiescent cells were stimulated to re-enter the cell cycle by addition of 10% FBS. At each indicated time point, cells were collected and washed with cold PBS (phosphate-buffered saline and fixed in 70% ethanol for greater than 15 min). Cells were then treated with RNase (50 µg/ml) in 1.0% sodium citrate at 37°C for 30 min and then stained with propidium iodine (50 µg/ml) for 15 min. DNA content was measured with a fluorescence-activated cell analyzer (FacScan flow cytometer, Becton Dickson, San Jose, CA). The percentage of cells in each phase of the cell cycle was determined using the WinList and ModFit programs (Verity Software House, Topsham, ME).

### Statistical Analysis

Statistical analysis was performed using GraphPad Prism V4.03 Software and the R V2.7.0 Statistical Computing Environment. A *p*-value less than 0.05 was considered statistically significant. Student’s *t*-test was used for most comparisons of SRPK1 RNA and protein expression vs clinicopathological parameters. The methods of Kaplan and Meier and of Cox were used to estimate median survival time and hazard ratios.

## Results

### SRPK1 Expression is Elevated in Certain Ovarian Carcinoma Cell Lines and Ovarian Tumors

**Figure 1:**
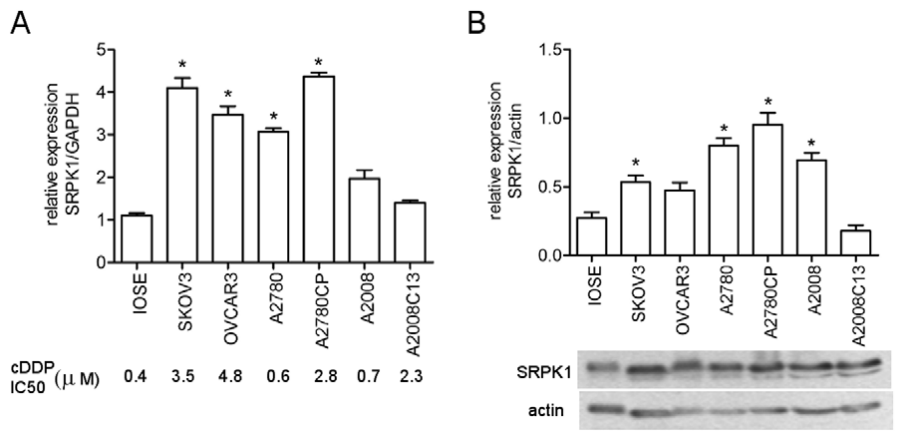
Expression of SRPK1 protein in ovarian cancer cell lines sensitive or resistant to cDDP. (A) Quantitative real time reverse-transcription polymerase chain reaction (RT-PCR) analysis of SRPK1 RNA level in 6 OVCa cells and IOSE cells. GAPDH was used for loading controls and normalization. Fifty percent growth inhibition concentration (IC_50_, µM) of cDDP for the OVCa cells was indicated lower panel). (B) Western blot analysis of SRPK1 protein in 6 OVCa cells and IOSE cells. The lower band in the blot probed with anti-SRPK1 antibody is probably proteolytic fragment of SRPK1. The blots were re-probed with actin which was used as a loading control and for normalization. Graph represents relative expression determined by densitometric measurement of the bands and is expressed relative to actin. Bar-graph shown is the average of 4 experiments. Overexpression of SRPK1 in OVCa was defined as greater than 2 fold over the mean of the IOSE samples and is marked with asterisks.

**Figure 2:**
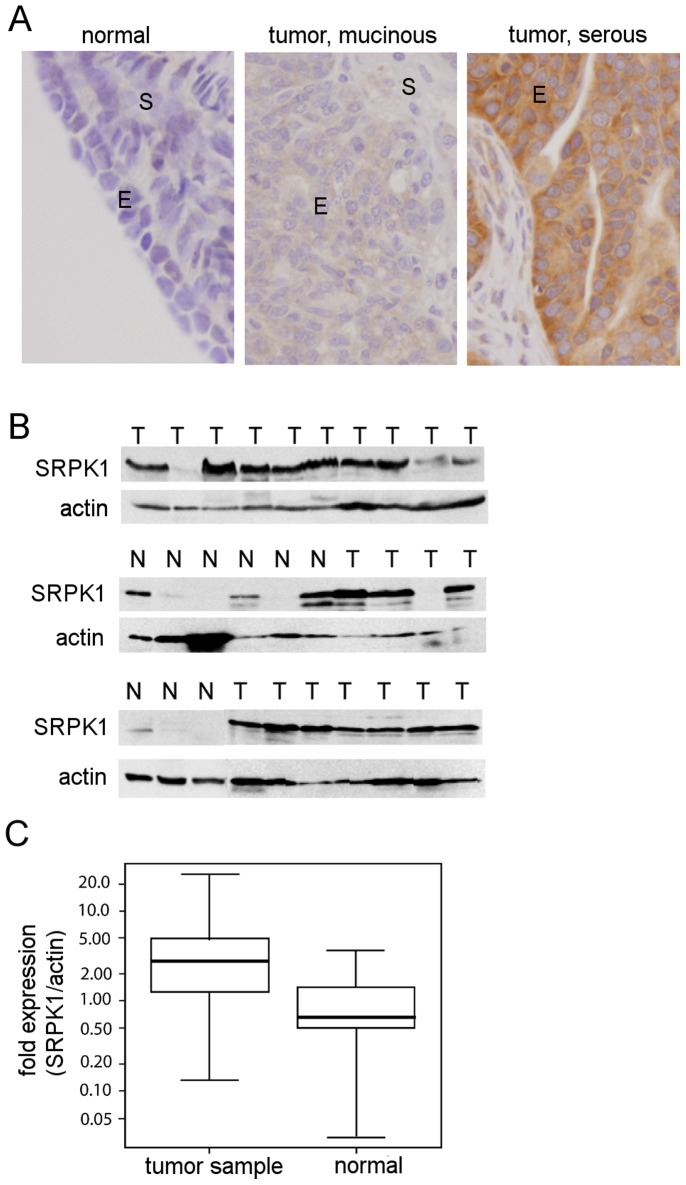
SRPK1 is overexpressed in 55% of ovarian tumor samples. (A) Immunohistochemical staining for SRPK1 protein expression in normal and tumor ovary tissue sections. The cells staining positively for SRPK1 antibody are brown. For negative control, the host-IgG was used (not shown). E, epithelium; S, stroma. (B) Western blot analysis of SRPK1 protein in 21 ovarian tumor samples (T), and 9 non-neoplastic ovarian tissue samples (N). The normal and tumor samples in the lowest panel were cropped from two different blots for the purpose of presentation. The lower bands in the blot probed with anti-SRPK1 antibody are probably proteolytic fragments of SRPK1. (C) Box-Whisker plot with SRPK1/actin fold expression derived from densitometric analysis of the bands in Western blot analysis. Whiskers encompass all the tumors (n = 47) or the normal samples (n = 9), boxes contain 50% of data, and the center lines in the boxes show medians. Mean expression was compared by Student’s *t*-test (p = 0.03).

We have previously found that inactivation of the yeast SR protein kinase Sky1p confers resistance to cisplatin [22]. However, the level of the human SRPK1 gene has been both positively and negatively correlated with resistance of tumor cells to treatment regimens containing platinum [18], [19], [20], [23]. We set out to further examine the relationship between the expression level of the SRPK1 gene and cDDP resistance in human ovarian cancer (OVCa). We first used a semi-quantitative RT-PCR (reverse-transcriptase polymerase chain reaction) analysis for SRPK1 RNA expression in 6 OVCa cell lines with differential cDDP sensitivity (Figure 1A, bottom panel). Some of which were at least 6 fold more resistance to cDDP than a non-transformed ovarian surface epithelial cell line (IOSE) which had been immortalized with the SV40 early genes [27]. The data show that there was no clear correlation between cDDP resistance phenotype and the RNA levels of these genes among these cell lines. However, SRPK1 mRNA level was elevated in 4 out of 6 cell lines, as compared with that of the IOSE cells (Figure 1A). We next assessed the SRPK1 protein expression in the above mentioned cell lines using Western blot analysis. The relative level of SRPK1 protein was determined by normalization with that of the housekeeping gene actin. Figure 1B shows that SRPK1 protein is also elevated in 4 out of 6 ovarian carcinoma cell lines (defined as >2-fold over the mean of the IOSE sample), albeit with slightly different pattern from that of the RNA. We noticed that the immortalized IOSE cells also express certain level of SRPK1. This is not surprising since others have also demonstrated that immortalization of ovarian epithelial cells, as compared with the non-immortalized normal human OSE cells, increases the expression of a splicing factor, polypyrimidine tract-binding protein [8]. Similar to the mRNA expression, we found no clear correlation between cDDP resistance phenotype and the SRPK1 protein level among these cell lines. Since gene levels in long term cultured cell lines may be altered, we next examined SRPK1 expression in archived OVCa tumor samples from OVCa patients who were treated with platinum-regimens after surgery. To assess the specific expression of SRPK1 protein in tumor epithelium at the cellular level, we performed immunohistochemical staining (IHC) on paraffin-embedded tissue sections. Figure 2A shows that SRPK1 staining was almost nonexistent in normal ovarian epithelium. In contrast, SRPK1 staining was readily detected in ovarian tumors, with staining intensity ranging from weak to strong, and present predominantly in the cytoplasm of epithelial cells. The cytoplasmic localization of SRPK1 in OVC tumor samples is similar to findings in tissue culture cells [15]. Since ovarian tumors are composed mostly of epithelial cells, the level of SRPK1 in protein homogenates from 47 frozen tumor and 9 non-neoplastic tissue samples was investigated using Western blot analysis. Figure 2B shows the immunobloting results from 21 tumor and 9 non-neoplastic tissue samples. Densitometric analysis of SRPK1 and actin levels was performed and the relative expression level of SRPK1 was normalized with the level of actin. We found that 26 out of 47 (55%) cases overexpressed SRPK1 (defined as greater than two-fold over the mean of the nine normal samples).

The majority of the 47 patients tested presented with grade 3 tumors (91%), at stage IIIC (76%), and with serous histology (85%). The median survival for all patients was 39.7 months {confidence interval (CI), 26.5-∞ months}, whereas the median disease-free survival, excluding patients with persistent/progressive disease after initial therapy, was 17.5 months (CI, 14.4–35.9 months). Using a Cox regression model we did not observe a clear correlation between SRPK1 level and overall (p = 0.26) or disease free (p = 0.62) survival in the ovarian cancer patients. SRPK1 levels were not correlated to histology, grade, or clinical response to cDDP-containing chemotherapy regimen. However, the mean relative fold expression of the SRPK1 protein was significantly different between tumor samples and normal samples (Figure 2C; independent samples t-test, p-value = 0.03). Thus, our data indicate that elevated level of SRPK1 protein expression is a frequent event in ovarian epithelial malignancies.

### Reducing the Level of SRPK1 by Short Hairpin RNA Enhances cDDP Cytotoxicity

**Figure 3:**
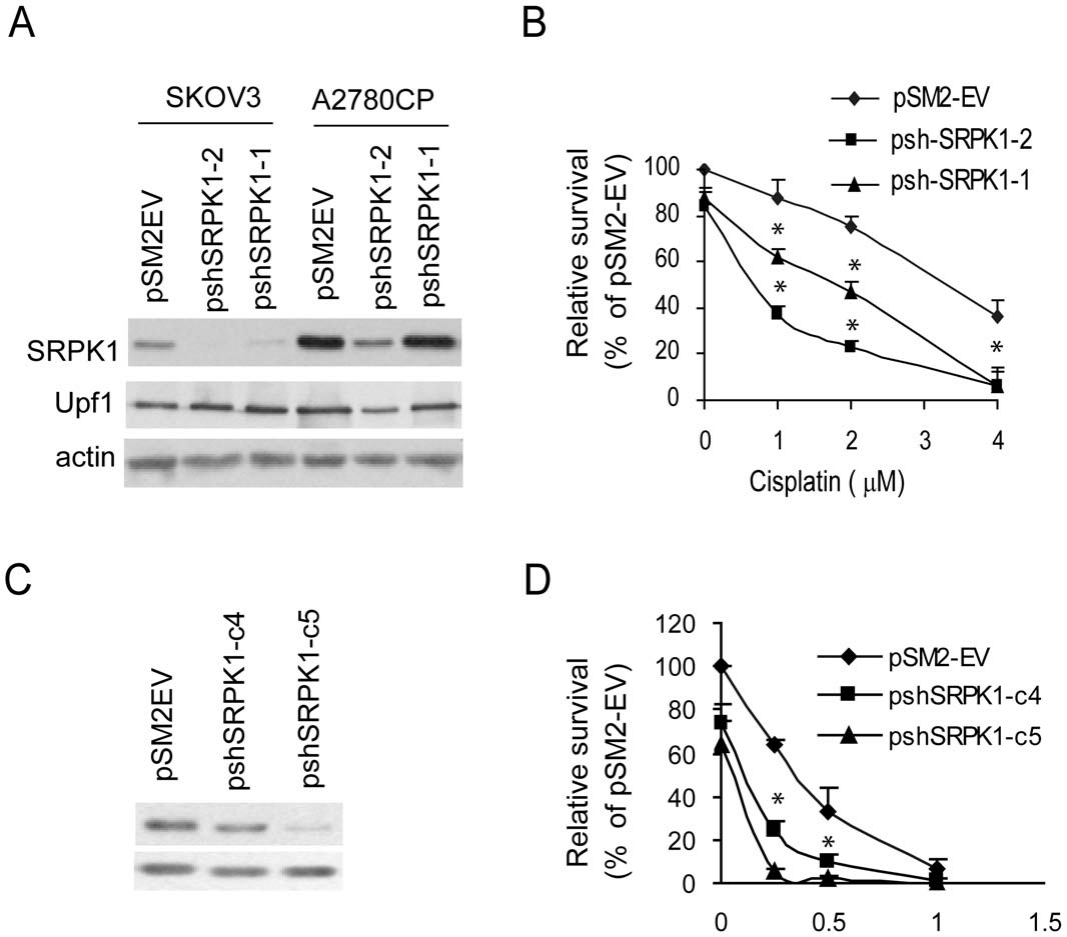
Forced inhibition of SRPK1 expression in ovarian cancer cell lines enhances sensitivity to cisplatin. Ovarian cancer cells were transiently (A) or stably (C) transfected with either the siRNA-encoding shSRPK1 plasmid or the empty vector (pSM2-EV). Protein levels of SRPK1, UPF1 and actin were determined by Western blot analysis. Representative blots from three independent experiments are shown. (B) and (D) SRPK1 knockdown enhances cisplatin cytotoxicity. (B) Cells (5×10^4^) were reseeded 24 h after transfection, treated with various concentrations of cisplatin for 48 hr and the number of surviving cells was analyzed by MTT assay. Survival (%) is expressed relative to non-treated pSM2-EV cells. (D) Stable transfectants (3×10^2^) were seeded in triplicate, treated with cisplatin for 24 hr and colony formation was assessed after 10–14 days. Data were analyzed with one way ANOVA and * indicates P<0.05; bars, SD.

It has been shown that disruption of SRPK1 expression by small interfering RNA increases apoptosis caused by cDDP in pancreatic, colon and breast cancer cell lines [18], [19]. To test whether knockdown of the SRPK1 gene in OVCa cells affects their sensitivity to cDDP, we targeted this gene at two positions in the kinase domain with two different constructs of short hairpin RNA (sh-SRPK1-1 and sh-SRPK1-2) in SKOV3 and A2780/CP cells, both of which are relatively more resistant to cDDP than the IOSE cells and some other OVCa cell lines (Figure 1A). As controls, the vector pSM2-EV was also transfected in both cell lines. Transient transfection (48 hr) of either construct specifically reduced the SRPK1 mRNA (data not shown) and protein level as confirmed by reprobing the Western blots with antibody against a non-related protein, Upf1, and actin was used as a loading control (Figure 3A). The inhibitory effect of the shSRPK1-2 construct is more significant than that of the shSRPK1-1. Using the formazan color (MTT) assay, Figure 3B shows that reducing SRPK1 protein in SKOV3 cells resulted in enhanced sensitivity to cDDP treatment (paired t-test and * indicates P<0.05). To further study the chemo-sensitizing effect of inhibiting SRPK1, we generated stable clones with different shRNA potencies in SKOV3 cells. We obtained several clones with reduced SRPK1 mRNA and protein as assessed by RT-PCR and Western blot analysis, respectively. Two of them are shown in Figure 3C where SRPK1 protein level was reduced to approximately 60% (pshSRPK1-c4, targeted by sh-SRPK1-2) or 20% (pshSRPK1-c5, targeted by sh-SRPK1-1) of the control pSM2-EV cells. SRPK1 levels in these clones correlate inversely with their cDDP sensitivity as determined by colony formation assay (Figure 3D). These data indicate that SRPK1 plays a role in modulating cDDP cytotoxicity *in vitro* and that targeting nucleotides 288 to 308 of the SRPK1 mRNA resulted in a stronger silencing effect.

### Knockdown of SRPK1 Expression Decreased Cell Proliferation and *in vitro* Tumorigenic Potential of SKOV3 Cells

**Figure 4:**
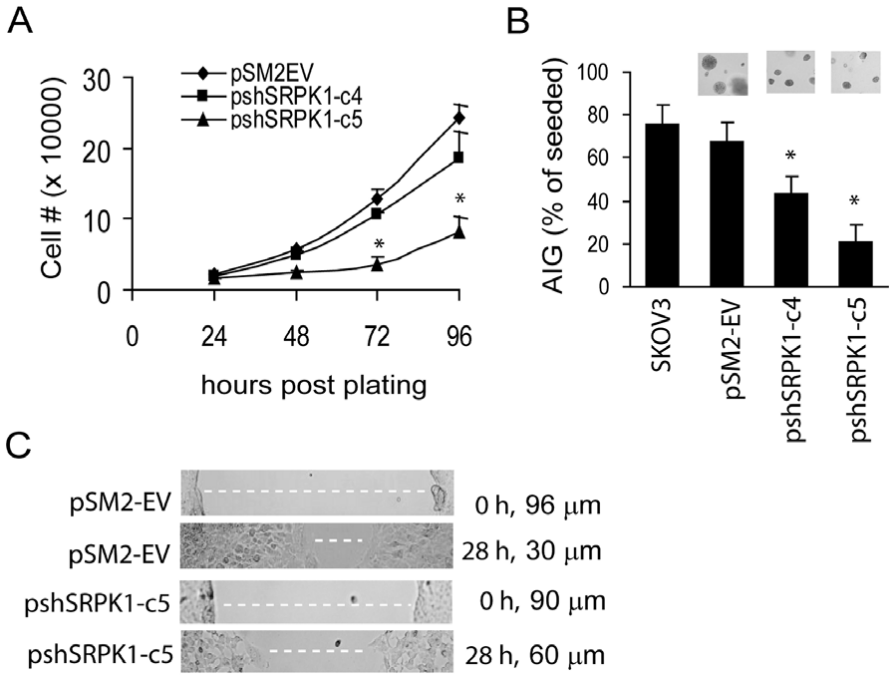
SRPK-knockdown ovarian cancer cells exhibit reduced cell proliferation, anchorage-independent growth and *in vitro* migration ability. (A) Cell proliferation assay. Cells (1×10^4^) were seeded in triplicate in a 24-well plate, trypsinized and counted in the presence of trypan blue at the indicated times. (B) Anchorage-independent growth assay. Cells (5×10^3^) were seeded in soft agar and colony number was determined 21 days later. Representative fields of the colonies in soft-agar plates are also shown. Data shown are the mean of three independent experiments and were analyzed with paired t-test; * indicates P<0.05, bars, SD. SKOV3 cells were seeded as a positive control. (C) *In vitro* wound healing assay. Confluent cells were scratched with a 200 µl-tip to generate straight line-gaps. Images of the gaps were taken at 0 and 28 hr and the width of the gaps (white dashed-lines) was measured under a light microscope to calculate the rate of cell migration. Representative images are shown on the left and relative migrating distances (wound closure) shown on the right were calculated from 20 areas from each plate. Data shown are from three independent experiments. *Asterisks* show significant differences compared with control (*P*<0.05).

Data shown in Figure 3D also indicate that cells with reduced SRPK1 expression by shRNA grew slower than the control pSM2-EV cells. They formed fewer colonies than the non-treated control group in the absence of cDDP treatment. To further test whether reducing SRPK1 expression inhibits ovarian cancer cell proliferation, the doubling time of pSM2-EV and pshSRPK1-c5 cells was compared. Cell proliferation rate was determined in exponentially growing cells by trypsinization and trypan blue dye exclusion at 24, 48, 72, and 96 hr post plating. Figure 4A shows that the doubling time of pshSRPK1-c5 is approximately 1.4 fold of the control pSM2-EV cells (31 hr vs. 22 hr). This was confirmed by MTT assay (data not shown). We next investigated the tumorigenic potential of SRPK1 using the anchorage-independent-growth (AIG) assay. SRPK1 knockdown and control cells were seeded in the soft agar and allowed to grow for 3 weeks. Figure 4B shows that both shSRPK1 clones produced fewer and smaller colonies in soft agar, suggesting a reduction in *in vitro* tumorigenicity. Cell motility is one of the factors that contribute to tumor cell invasion. To test whether cell migration ability is compromised in the SRPK1 knockdown cells, an *in vitro* cell migration (wound healing) assay was performed. Figure 4C shows that the average migration rate for shSRPK1-c5 cells (5.8±0.81 unit) was approximately 60% of that for the control pSM2-EV cells (9.9±1.5 unit). Together, our data indicate that SRPK1 contributes to cell proliferation, *in vitro* cell migration and tumorigenic potential of SKOV cells.

### Knockdown of SRPK1 Expression Reduced Phosphorylation of Certain SR Proteins and MAPK/AKT Proteins

**Figure 5:**
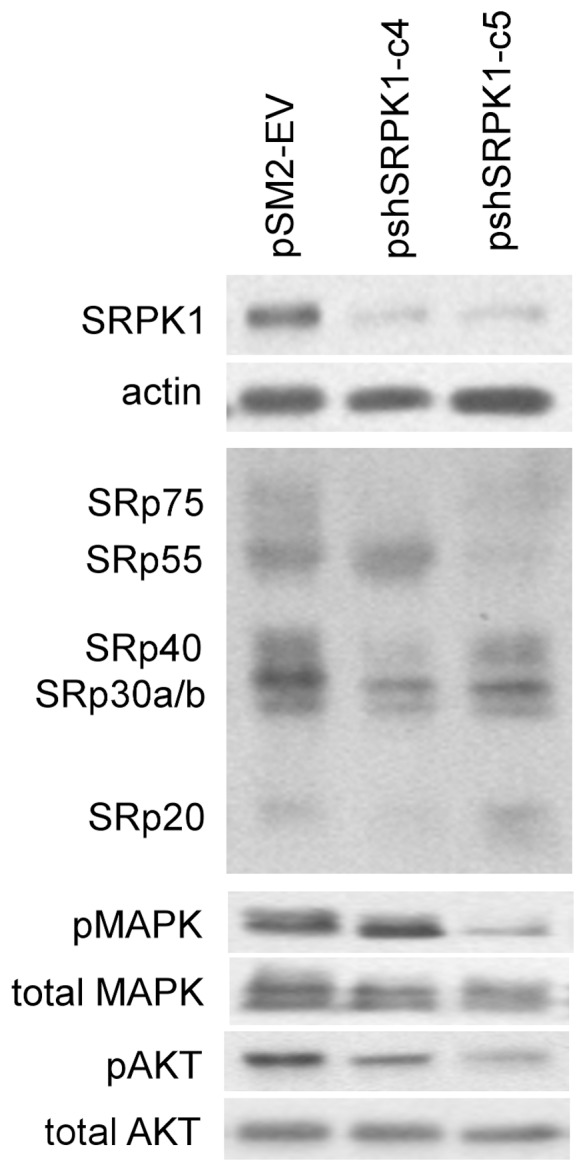
Phosphorylation patterns of SR-proteins and MAPK42/44 and AKT proteins are altered in SRPK1-knockdown SKOV3 cells. Western blot analysis was performed on lysates prepared from SKOV3-derived pSM2-EV cells and two stable SRPK1-knockdown clones. Antibodies were used to detect the phosphorylated SR proteins (mAB104), MAPK42/44 (Thr^202^/Tyr^204^), and AKT (Ser^473/^Thr^308^) as well as total protein levels of MAPK42/44, and AKT.

SR proteins are the direct targets of SRPK1 [15]. The phosphorylation pattern of these SR splicing factors is expected to be affected in SRPK1 knockdown cells. To test this prediction, Western blot analysis using a pan antibody recognizing a phospho-specific epitope common to multiple SR proteins [29] was performed. As expected, reduced SRPK1 expression resulted in decreased levels of phosphorylation of certain SR proteins in SKOV3 cells (Figure 5, middle panel). It is interesting to note that different SR proteins are affected between shSRPK1-c4 and shSRPK1-c5 clones. For example, the level of SRp55 was reduced much more in shSRPK1c5 than that in shSRPK1-c4 cells. The opposite is true for the SRp40 and SRp20. The differential knockdown of SRPK1 observed in these two clones (Figure 5, top panel) may account for the differences in substrate phosphorylation. The level of the SRPK1 enzyme may determine its substrate recognition and catalytic activity. The differences of the SRPK1 level between shSRPK1-c4 and shSRPK1-c5 clones are also reflected in cell proliferation and sensitivity to cisplatin (see below) affected by SRPK1 downregulation.

It is well known that activation, i.e. elevated level of phosphorylation, of MAPK (mitogen-activated protein kinase) and AKT signaling pathways is involved in many malignancies (29, 30) and that these pathways regulate pre-mRNA processing [31], [32]. The phosphorylation pattern of the two key transducers of proliferation signals, p44/42-MAPK (ERK1 and ERK2) and AKT was analyzed in the SRPK1 knockdown cells. Figure 5 shows that pshSRPK1-c4 and pshSRPK1-c5 cells had reduced levels of phosphorylated p44/42-MAPK (p-MAPK) and AKT (p-AKT) proteins. In contrast, the total amount of MAPK and AKT proteins was not affected by the inhibition of SRPK1 expression. The reduced levels of phosphorylated p44/42-MAPK and AKT correlated with slower growth of the SRPK1 knockdown cells (Figure 4). These data suggest that SRPK1 plays a role in regulating the level of activated forms of MAPK and/or AKT proteins.

### SRPK1-knockdown Cells Exhibit Slower Cell Cycle Progression

**Figure 6:**
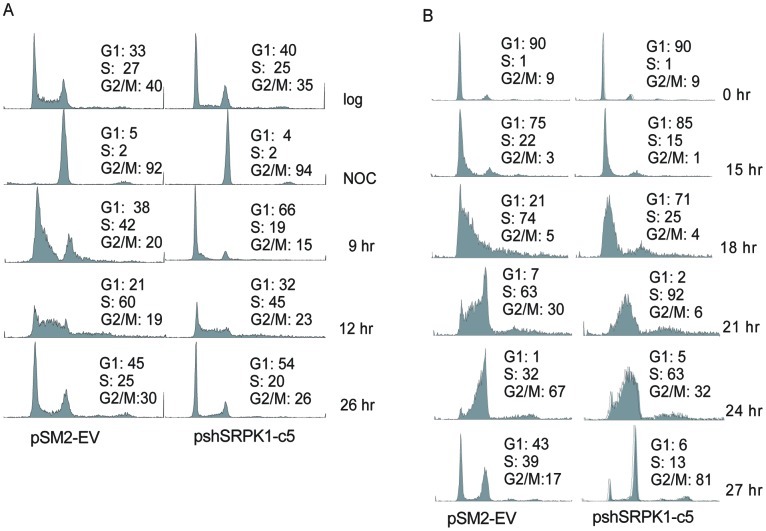
Effects of SRPK1-knockdown on cell cycle progression in SKOV3 cells. (A) Cell cycle progression from G2/M-arrest. Cells at 80% confluence were arrested in G2/M phases with nocodazole (NOC, 600 ng/ml) for 12 hr, washed with phosphate buffered saline (PBS), and released into medium without drug. Cells were collected at the indicated times and subjected to propidium iodine staining and flow cytometric analysis. Representative data obtained from two independent experiments are shown. (B) Cell cycle progression from G0/G1-arrest. Cells at 100% confluence were incubated in medium without serum. Quiescent cells were activated with 10% FBS-containing medium. Cells were collected every 3 hr for flow cytometric analysis as described above. The percentage of cells in G1, S, and G2/M was determined using Modfit program and a histogram graph was generated using the WinList program (Verity Software House). Representative data obtained from two independent experiments are shown.

Data described above indicate that inhibition of SRPK1 leads to slower cell growth and reduced phosphorylation of certain MAP kinases, which are major regulators of cell cycle progression. We hypothesized that the slower proliferation rate of shSRPK1-knockdown cells was due to an alteration in cell cycle progression. We thus evaluated the cell cycle distribution in the shSRPK-c5 clone and the pSM2-EV cells using two different cell synchronization methods and fluorescence activated cell sorter (FACS) analysis. First, cells were arrested in G2/M phase with nocodazole. After releasing into cycle, cells were collected every 3 hr for the first 12 hr and then at the 26-hr time point for analysis of cell cycle distribution. Figure 6A shows that shSRPK1c5 cells exhibited a longer G1 phase compared with that of the pSM2-EV cells. This is evident in the FACS profile at 9 hr after release of cells from G2/M phase. At this time point, 66% of the pshSRPK1-c5 cell population, compared with 38% of the pSM2-EV cell population, was in G1 (Figure 6A). This result was confirmed using a different synchronization method. Cells were arrested in G0/G1 phase by serum starvation and the quiescent cells were released into the cell cycle by addition of serum. Figure 6B also shows that shSRPK1-c5 cells exhibited a longer transition from G1 into S phase as compared with pSM2-EV cells. This is evident in the FACS profile at 18 hr after release of cells from serum starvation. At this time point, 25% of the pshSRPK1-c5 cell population, compared with 74% of the pSM2-EV cell population, was in S phase (Figure 6B). The effects of SRPK1-knockdown on the progression through S and G2/M phases were not significant as determined by both synchronization methods. Thus these results indicate that SRPK1 regulates the activities of proteins that mediate cell cycle progression through G1 to S phases, thereby regulating ovarian cancer cell growth.

To test the specificity of the shRNA knockdown effects, i.e. whether the phenotypes described above are related to an off-target effect of the shRNAs used, we performed a rescue experiment by reintroducing into the pshSRPK1-c5 cells a SRPK1-expressing construct carrying three silent mutations in the shRNA-targeting sequences (see Material and Methods). A transient re-expressing of a functional SRPK1 in the knockdown cells was observed, judging from the phosphorylation pattern of the SR proteins (data not shown). However, the exogenous SRPK1 protein level was not maintained during our attempt to select stable clones. It is likely that the silent mutation sequences were still recognized by the shSRPK1-RNA. Thus these results suggested that the phenotypes of the pshSRPK1-c4 and pshSRPK1-c5 cells were a shRNA-target-specific effect.

## Discussion

It is estimated that about 15% of disease-causing mutations in human genes involve mis-regulation of alternative splicing [33]. The experiments reported here show that SRPK1, a major regulator of the alternative splicing process, is elevated in more than 50% of epithelial ovarian tumor samples. Our data together with previous reports [18], [19] indicate that elevated SRPK1 expression is common in epithelial cancers. Additionally, our findings show that forced inhibition of SRPK1 expression leads to (i) reduced phosphorylation of its direct substrates, SR proteins, and MAPK and AKT proteins, and (ii) decreased cell proliferation, slower cell cycle progression and decreased *in vitro* tumorigenic potential. Furthermore, we demonstrated that inhibition of SRPK1 enhanced sensitivity of OVCa cells to cDDP. These results suggest that therapeutic targeting of SRPK1 in combination with conventional chemotherapeutic agents can increase the efficacy of ovarian cancer therapy.

Although SRPK1 is ubiquitously expressed in many tissue types, it is highly expressed only in testicular germ cells and is thought to be a cancer/testis-like antigen which usually has restricted tissue distribution [21]. Elevated levels of SRPK1 have been reported in different types of malignancies [16], [18], [19], [21]. Our results using immunohistochemical staining on a limited number of samples provided a strong hint that SRPK1 is overexpressed in ovarian tumors, compared with normal ovarian epithelium. This notion was further confirmed by Western blot analysis that demonstrated that more than 50% (26 out of 47) of ovarian tumors overexpress this gene. This raises the possibility that SRPK1 could be a useful marker for the diagnosis of OVCa. Other reports using immunohistochemistry have found that the level of SRPK1 protein correlates with the grade of breast (n = 12) and colon (n = 15) cancer [19]. While our Western blot analysis indicated that the relative level of SRPK1 expression did not correlate with any clinicopathologic features of the patients, its cancer/testis-like expression pattern, along with the relatively high rate of overexpression in ovarian tumors prompted us to determine its potential biological role in this disease. Since it is possible that tumor heterogeneity in the tissue lysates used in Western blotting may confound the results, future studies using immunohistochemical staining on an ovarian tumor tissue microarray to compare SRPK1 levels in a larger collection of ovarian tumors with normal tissue samples are warranted to clearly delineate the impact of SRPK1 expression on clinicopathologic characteristics of ovarian cancer.

Some alternative splicing factors have been found to be elevated in a variety of cancers [7], [34], [35] pointing to the important roles played by the catastrophic effects of aberrant pre-mRNA splicing. For example, elevated levels of splicing factors, such as PTB and SRp20 [36], [37], have been demonstrated in ovarian cancer. Other SRPK1 substrates such as, SF2/ASF and SC35 (SRp30b) splicing factors have also been shown to be overexpressed in several cancer types [7]. The importance of these factors is manifested by the fact that they also function in mRNA transport, stability, and translation [22]. Overexpression of SRPK1 is thought to result in constitutive phosphorylation of SR proteins thereby inhibiting their de-phosphorylation and consequent splicing reaction [14], [38]. Our data indicate that inhibition of SRPK1 reduces the level of phosphorylation of several SR proteins (Figure 5). Conceivably, this would affect many splicing events and the level of transcripts and change the repertoire of cellular proteins. Results shown here and by others indicate that SRPK1 levels regulate phosphorylation (activation) of the MAPK and AKT pathways. Both pathways transduce signals to promote cell proliferation and regulate cell cycle progression [39], [40]. Indeed we found that decreased expression of SRPK1 in OVCa cells leads to known MAPK- or AKT-regulated events, namely, reduced growth rate (Figure 4) and slower cell cycle transition from G1 to S phase (Figure 6). The changes in splicing fidelity elicited by SRPK1 down-regulation in breast and colon tumor cells have been shown to lead to production of aberrant MAP2K2 transcripts and protein, which in turn may reduce the availability of this kinase to phosphorylate its targets MAPK1/2 [19]. In agreement with our findings, recent reports also show that siRNA knockdown of one of the splicing cofactors (SON) decreased cell cycle progression [41]. Our results that SRPK1 also regulates cell migration and tumorigenic potential *in* *vitro* suggest that some tumor suppressor genes, oncogenes and/or cell adhesion molecules are targets of SRPK1-mediated splicing events and other transcriptional processes. Others have also found several abnormal splice variants of tumor suppressors and oncogenes in cells overexpressing the SF2/ASF splicing factor, one of the targets of SRPK1 [7]. Nevertheless, more studies are required to delineate the detailed mechanisms by which SRPK1 mediates cell transformation. For example, a genome-wide splice variant analysis on the parental and the SRPK1-knockdown cells should help to identify the diverse set of genes involved in the SRPK1-regulated signaling network.

Whether a high level of SRPK1 gene is directly associated with resistance to cDDP treatment in human cancer cells remains controversial. Although others have found an inverse association between SRPK1 expression and cDDP or oxaliplatin sensitivity in retinoblastoma [42], our clinico-pathologic correlative studies did not show a relationship between cisplatin sensitivity and the level of SRPK1 expression in archived tumor samples. In addition, while results shown here and elsewhere [19] demonstrated that forced inhibition of SRPK1 using shRNA constructs sensitizes cancer cells to cisplatin treatment *in vitro* (Figure 3), Schenk et al. have found that down-regulation of SRPK1 with antisense oligodeoxynucleotides in A2780 cells confers resistance to cisplatin [43]. One possible explanation of the observed discrepancy is that shSRPK1 construct alone induces cell death (Figure 3 and [19]) whereas the antisense-SRPK1 used by the other study does not show cytotoxic effect [43]. It is also possible that the effects of inhibiting SRPK1 expression on cisplatin toxicity depend on the baseline levels of SRPK1 and its target genes in the particular cell line. In addition, the influence of other genetic contexts of each cell line on drug sensitivity cannot be excluded. Furthermore, both SRPK1 overexpression and under expression cause aberrant phosphorylation of target splicing factors [14], [15]. Thus, unlike the simpler yeast models [22], [23], the relationship of SRPK1 function and cDDP resistance in human cancer cells requires further clarification.

In summary, our results demonstrate that a significant proportion of epithelial ovarian cancers overexpress SRPK1. Whether SRPK1 overexpression is the cause or a result of ovarian malignancy requires further study. For example, it remains to be determined whether overexpression in non-transformed cells will result in transformation *in vitro* and *in vivo*. Our findings also suggest that modulating SRPK1 levels to affect splicing factor activity and subsequent translation of mRNAs and expression of genes associated with growth control provides an opportunity to treat the abnormal growth of ovarian cancer cells.
